# Deletions on Chromosome Y and Downregulation of the *SRY* Gene in Tumor Tissue Are Associated with Worse Survival of Glioblastoma Patients

**DOI:** 10.3390/cancers13071619

**Published:** 2021-03-31

**Authors:** Małgorzata Łysiak, Anja Smits, Kenney Roy Roodakker, Elisabeth Sandberg, Anna Dimberg, Munila Mudaisi, Charlotte Bratthäll, Michael Strandeus, Peter Milos, Martin Hallbeck, Peter Söderkvist, Annika Malmström

**Affiliations:** 1 Department of Biomedical and Clinical Sciences, Linköping University, 58185 Linköping, Sweden; munila.mudaisi@regionostergotland.se (M.M.); peter.milos@regionostergotland.se (P.M.); martin.hallbeck@liu.se (M.H.); annika.malmstrom@regionostergotland.se (A.M.); 2 Department of Neuroscience and Physiology, Clinical Neuroscience, Sahlgrenska Academy, University of Gothenburg, 41345 Gothenburg, Sweden; anja.smits@neuro.gu.se; 3 Department of Neuroscience, Neurology, Uppsala University, University Hospital, 75185 Uppsala, Sweden; kenney.roodakker@radboudumc.nl (K.R.R.); elisabeth.sandberg@sll.se (E.S.); 4 Institute of Immunology, Genetics and Pathology, Uppsala University, 75185 Uppsala, Sweden; anna.dimberg@igp.uu.se; 5 Department of Oncology in Linköping, Linköping University, 58185 Linköping, Sweden; 6 Department of Oncology, District Hospital, 39185 Kalmar, Sweden; charlotte.bratthall@regionkalmar.se; 7 Department of Oncology, Ryhov Hospital, 55185 Jönköping, Sweden; michael.strandeus@rjl.se; 8 Department of Neurosurgery in Linköping, Linköping University, 58185 Linköping, Sweden; 9 Department of Clinical Pathology, Linköping University, 58185 Linköping, Sweden; 10 Department of Advanced Home Care, Linköping University, 58185 Linköping, Sweden

**Keywords:** glioblastoma, Y chromosome, LOY, SRY, sex bias, survival

## Abstract

**Simple Summary:**

Glioblastoma (GBM) is one of the most common and most aggressive brain tumors with higher prevalence among men than women. Loss of chromosome Y (LOY) in the peripheral blood cells has been associated with increased risk of developing cancer. However, there is a lack of data about LOY in GBM tumor tissue and the potential impact on patients’ prognosis. Through droplet digital PCR (ddPCR) analysis of 10 markers spread throughout chromosome Y in 105 male GBM patients, we were able to identify deletion of *SRY* gene as a factor strongly correlating with reduced overall survival. This finding was later corroborated by the analysis of GBM gene expression data collected in TCGA, showing correlation between decreased *SRY* expression and shortened overall survival.

**Abstract:**

Background: Biological causes of sex disparity seen in the prevalence of cancer, including glioblastoma (GBM), remain poorly understood. One of the considered aspects is the involvement of the sex chromosomes, especially loss of chromosome Y (LOY). Methods: Tumors from 105 isocitrate dehydrogenase (IDH) wild type male GBM patients were tested with droplet digital PCR for copy number changes of ten genes on chromosome Y. Decreased gene expression, a proxy of gene loss, was then analyzed in 225 IDH wild type GBM derived from TCGA and overall survival in both cohorts was tested with Kaplan–Meier log-rank analysis and maximally selected rank statistics for cut-off determination. Results: LOY was associated with significantly shorter overall survival (7 vs. 14.6 months, *p* = 0.0016), and among investigated individual genes survival correlated most prominently with loss of the sex-determining region Y gene (SRY) (10.8 vs. 14.8 months, *p* = 0.0031). Gene set enrichment analysis revealed that epidermal growth factor receptor, platelet-derived growth factor receptor, and MYC proto-oncogene signaling pathways are associated with low SRY expression. Conclusion: Our data show that deletions and reduced gene expression of chromosome Y genes, especially SRY, are associated with reduced survival of male GBM patients and connected to major susceptibility pathways of gliomagenesis.

## 1. Introduction

Gliomas are a mixed group of primary brain tumors, which in the last couple of years gained improved classification thanks to the inclusion of molecular markers into the diagnostic process [1]. Glioblastoma (GBM) remains infamously the deadliest and the most frequent among glioma entities, with a median overall survival (OS) of merely 1.2 years [2]. Similar to many other types of cancer, GBM is more frequent among males, with a male to female ratio of 1.6:1, as reported for the USA patients diagnosed with GBM between 2011 and 2015 [3,4]. Even more striking is the fact that females have better OS than men [5]. However, sex disparity and its potential influence on the disease have been overlooked for many years, both in cancer, in general, and in glioma research, specifically [6]. In the age of tailoring individual treatments, it will be important to identify molecular mechanisms causing such an imbalance. In fact, studies based on the data from The Cancer Genome Atlas (TCGA) have provided some insight already. Transcriptomes of gliomas are not clearly influenced by the sex of patients; however, the existing differences could be predominantly attributed to the sex chromosomes [7,8].

Despite the first reports about aneuploidy of sex chromosomes in brain tumors already decades ago [9,10,11], loss of chromosome Y (LOY) was commonly considered a physiological process in aging men [12,13,14], accelerated by smoking [15] and associated with increased risk of Alzheimer disease [16]. Renewed interest in the “genetic wasteland” of chromosome Y came with LOY associations to cancer [12,17,18,19], even though not all studies could confirm the relationship [13].

While available reports focus mainly on the somatic mosaicism of the entire chromosome Y and the association with disease, we opted for copy number (CN) analysis of selected genes as a possible approximation of the event with the advantage of studying single gene influence on the patients’ OS. We hypothesized that LOY influences the OS of male GBM patients, thus contributing to the observed sex bias. Here, we present the results that tie CN changes of genes located on the male sex chromosome to GBM OS.

## 2. Materials and Methods

### 2.1. Study Population

The study was approved by the Regional Ethics Committee, Linköping, Sweden. Patients eligible for the study were adult males with primary GBM, who all received temozolomide concomitant with radiotherapy and signed informed consent for the study protocol or were deceased at the time of sample collection without direct informed consent. Blood and tumor material from the southeast Sweden region were collected between 2008 and 2016 in a biobank at Linköping University Hospital. Tumor cells were enriched from marked areas on formalin-fixed paraffin-embedded (FFPE) tissue sections evaluated by an experienced pathologist and manually microdissected for DNA extraction. Depending on the material, Maxwell 16 FFPE Tissue LEV DNA Purification Kit (Promega) or Maxwell 16 LEV Blood DNA Kit (Promega, Madison, WI, USA) was used. After extraction, DNA was stored at −80 ˚C. Out of 113 identified tumor samples, the quality and quantity of DNA from 105 samples was suitable for further analysis. Clinicopathological data of the patients are summarized in Table 1. In accordance with the WHO classification [1], tumor samples were tested for isocitrate dehydrogenase 1 and 2 (IDH1/2) mutations with Sanger sequencing (described below) and all were negative. Matched blood samples were available for 61 of the patients.

The second cohort used in this study was derived from TCGA collection. Clinicopathological information as well as the processed transcriptome data of 119 male primary GBM, IDH wild type, were accessed through the GlioVis portal [20,21] (Supplementary Materials, Table S1). The relative mRNA expression values of specific genes on the Y chromosome and full transcriptome data were used in the following analyses. SRY mRNA expression data from TCGA for lower grade astrocytomas (grades II and III, IDH-mutated, 1p-19q intact) were also downloaded through the GlioVis portal. This group consisted of 83 male samples.

### 2.2. IDH1/2 and MGMT Analysis

Tumor DNA was amplified by PCR using MyTaq polymerase (Bioline, Cincinnati, OH, USA), with the annealing temperature of 57 °C, using primers: forward 5′-CAAAAATATCCCCCGGCTTG and reverse 5-ACATGCAAAATCACATTATTGCC for IDH1. The IDH2 exon 4 was amplified using primers: forward 5′- GGGGTTCAAATTCTGGTTGA, reverse 5′- CTAGGCGAGGAGCTCCAGT for the primary reaction and AAACATCCCACGCCTAGTCC together with the same reverse primer in the nested PCR, both with annealing temperature of 56 °C. All primers were designed with the online tool Primer3 [22]. Nested PCR was used due to the degradation of DNA extracted from FFPE tissue. PCR products were labeled according to the BigDye terminator v3.1 (Applied Biosystems, Foster City, CA, USA) protocol and separated in the ABI 3500 Genetic Analyzer (Applied Biosystems, Foster City, CA, USA). To assess O-6-methylguanine-DNA methyltransferase (MGMT) promoter methylation status, tumor DNA was first converted with EpiTect Bisulfite Kit (Qiagen, Hilden, Germany) and then used according to the Therascreen MGMT Pyro Kit (Qiagen, Hilden, Germany). Pyrosequencing was performed on PyroMark Q24 System (Qiagen, Hilden, Germany) and the cut-off for an unmethylated MGMT fraction was set to < 9 %.

### 2.3. Droplet Digital PCR (ddPCR)

**Figure 1:**
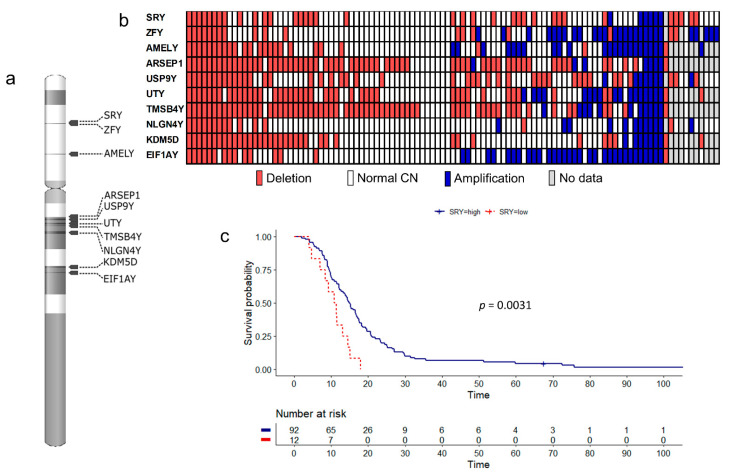
The ddPCR was used to test for copy number (CN) changes on chromosome Y, using 10 markers spread throughout both arms (**a**). We obtained CN results for all markers in 94 samples, detecting deletions (red; cut-off value 0.8), as well as amplifications (blue; cut-off value 1.2) (**b**). By applying maximally selected rank statistics analysis to the CN results (Supplementary Materials, Figure S1), we were able to choose the best deletion cut-off values, associated with shorter OS. Deletions of SRY (**c**) had significant association with shorter OS (10.8 vs. 14.8 months). For 11 samples results were not obtained for one or more genes.

Ten genes on chromosome Y—sex determining region Y (SRY), zinc finger protein Y-linked (ZFY), amelogenin Y-linked (AMELY) on arm p of the Y chromosome and arylsulfatase L pseudogene 1 (ARSEP1), ubiquitin specific peptidase 9 Y-Linked (USP9Y), ubiquitously transcribed tetratricopeptide repeat containing, Y-Linked (UTY), thymosin beta 4 Y-linked (TMSB4Y), neurologin 4 Y-linked (NLGN4Y), lysine demethylase 5D (KDM5D), and eukaryotic translation initiation factor 1A Y-linked (EIF1AY) on the q arm (Figure 1a)—were tested for CN alterations in tumor DNA with ddPCR. Markers were chosen based on the feasibility of designing specific primers and probes, due to the abundance of repetitive regions on chromosome Y and presence of homologous genes on chromosomes Y and X. The selected genes were also chosen to cover both chromosome arms. Each marker was tested separately. The DNA (10 ng) was mixed with SuperMix for Probes (no dUTP) (BioRad, Hercules, CA, USA) and probes specifically detecting the gene of interest (labeled with the fluorochrome FAM) and the reference gene (labeled with fluorochrome HEX) in ratios as suggested by the ddPCR protocol. The ddPCR method uses Poisson distribution to estimate the number of DNA copies per µL of reaction. Detailed information about probes and specific annealing temperatures can be found in Supplementary Materials, Table S2. Droplets were generated in the Automated Droplet Generator, and after PCR, according to the protocol from the ddPCR’s manufacturer, signal detection was carried out in the QX200 Droplet Reader (BioRad, Hercules, CA, USA). Dosage of SRY was tested also in 61 tumor-matched blood samples. DNA extracted from blood required addition of 1 µL of restriction enzyme HaeIII (Thermo Scientific, Waltham, MA, USA) to the reaction mix without any further changes in the protocol. As a reference gene for all the assays, AP3B1 with two stable copies located on chromosome 5 was chosen. Gene dosage was measured as the ratio of FAM positive droplets to HEX positive droplets, multiplied by 2.

### 2.4. Statistical Analysis

Gene dosage and TCGA mRNA expression results of selected markers extracted from the transcriptome data were tested for correlation with survival of the patients. Prior to the Kaplan–Meier survival analysis with log rank test, samples were dichotomized using maximally selected rank statistics for cut-point estimation [23]. The analysis assumed minimum 10% of samples present in one of the two groups and significance level of 95%. Packages maxstat and survminer in R 3.6.0 in RStudio version 1.3 were used to perform calculations and create the survival curves. Multivariate analysis was performed using the Cox proportional hazards model in IBM SPSS Statistics version 26. Comparison of the mean gene expression of SRY was tested with the Mann–Whitney U test with the significance level 0.05 also using IBM SPSS software.

### 2.5. Gene Set Enrichment Analysis (GSEA)

TCGA transcriptome data profiled on the Affymetrix HT Human Genome U133A microarray platform were used in the Gene Set Enrichment Analysis (GSEA) [24,25]. Samples were divided into low and high SRY mRNA expression groups based on the cut-off value estimated with maximally selected rank statistics (samples with expression 3.3867 (log2) or lower were included in the low expression group). Analysis was done using the 4.1.0 GSEA release with the default settings and included 9 main MSigDB gene sets collections with their subsets.

## 3. Results

### 3.1. Copy Number Alterations

CN changes on chromosome Y were tested with ddPCR using tumor DNA from 105 male IDH wild type GBM patients who underwent chemoradiotherapy after biopsy or surgical removal of tumor. Mean age of the patients was 57 years. Out of the 10 tested genes spread throughout the chromosome (Figure 1a), all showed variation in the number of copies present (Figure 1b). In 11 samples, we were unable to obtain results from all the markers, but all samples were included in the further single gene survival analysis. Samples with missing data for at least one marker were excluded from the LOY analysis. We observed complete and fractional deletions, as well as amplifications of genes. After normalization with the reference gene, number of copies of the gene of interest is given as a value with 95% confidence interval. The cut-off of 0.8 allowed for detection of fractional deletions with the higher limit of the 95% confidence interval always being below 2. Considering tumor heterogeneity, that would indicate a theoretical loss of a gene in 20% of cells of the tumor bulk. With this assumption, the most and least frequently deleted genes were TMSB4Y (64/96 samples; 66.7%) and NLGN4Y (14/102; 13.7%), respectively (Figure 1b). Only six samples harbored deletion of all markers, indicating complete LOY in the fraction of tumor cells. Amplification, assumed to occur whenever the lower limit of the 95% confidence interval was above 2 and gene copy number was 1.2 or higher (reflecting amplification in 20% of cells), was less common than a deletion. SRY was the least affected by amplification (6/104 samples; 5.8%), while EIF1AY was the most frequently amplified gene (31/96; 32.3%). Amplification of all 10 markers was found in four samples. Only six samples did not carry any chromosome Y dosage changes in any of the markers. For most of the samples, observed variations in the CN between markers were scattered, rarely affecting all adjacent genes. Nonetheless, in 44 % (42/95), including samples with LOY, we found a cluster consisting of three neighboring and co-deleted genes: ARSEP1, UTY, and TMSB4Y (Figure 1b).

### 3.2. Influence of Gene Dosage Alterations and mRNA Expression on Survival

**Figure 2:**
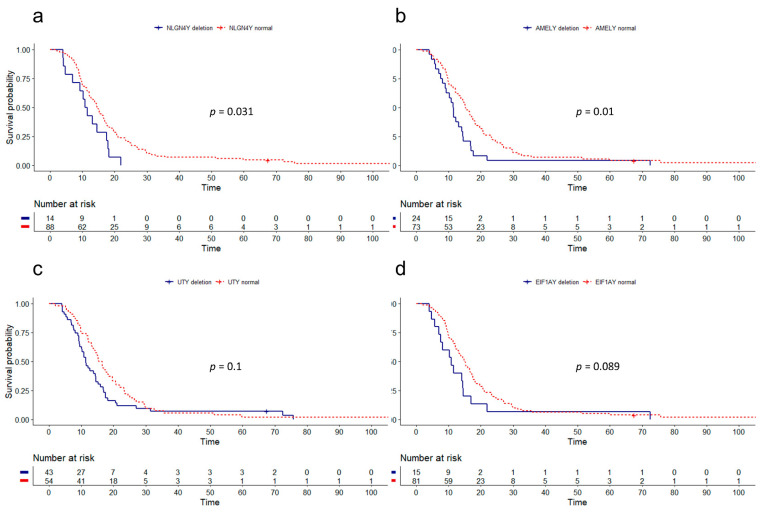
The log-rank Kaplan–Meier survival curves for genes (**a**) NLGN4Y (10.8 vs. 14.5 months, *p* = 0.031), (**b**) AMELY (11.5 vs. 15.2 months, *p* = 0.01), (**c**) UTY (11.5 vs. 15.3 months, *p* = 0.1), and (**d**) EIF1AY (10.8 vs. 15.1 months, *p* = 0.089) after re-analysis with the ddPCR CN cut-off value of 0.8.

Before survival analysis, CN results of each marker were used in the maximally selected rank statistics analysis to determine the cut-off value that would lead to the best separation of survival curves (Supplementary Materials, Figure S1). Dichotomization of patients according to the best cut-off for SRY (CN = 0.597) revealed the most significant difference in OS (*p* = 0.0031) (Figure 1c). Median survival of males with SRY deletion in at least 60% of the DNA was 4 months shorter than the OS of the remaining patients (10.8 vs. 14.8 months). Median OS also differed significantly in patients divided by the best CN cut-off value for NLGN4Y (CN = 0.936) (12.3 vs. 16.6 months, *p* = 0.0069), AMELY (CN = 0.836) (11.5 vs 15.3 months, *p* = 0.0081), UTY (CN = 0.97) (12.6 vs. 18.5 months, *p* = 0.024), and EIF1AY (CN = 0.95) (11.5 vs. 15.2 months, *p* = 0.0071) (Supplementary Materials, Figure S1a–e). However, these cut-off values exceeded our primary theoretical cut-off value of 0.8, assumed to detect deletion present in at least 20% of the DNA. Therefore, we re-analyzed the four genes (NLGN4Y, AMELY, UTY, and EIF1AY) using the arbitrary CN value of 0.8. With this cut-off, deletions of AMELY and NLGN4Y correlated with a shorter OS (11.5 vs. 15.2 months, *p* = 0.01; 10.8 vs. 14.5 months, *p* = 0.031, respectively) (Figure 2). In five cases (ZFY, USP9Y, ARSEP1, TMSB4Y, and KDM5D), no significant correlation with survival was detected (*p* > 0.05) (Supplementary Materials, Figure S1f–j).

**Figure 3:**
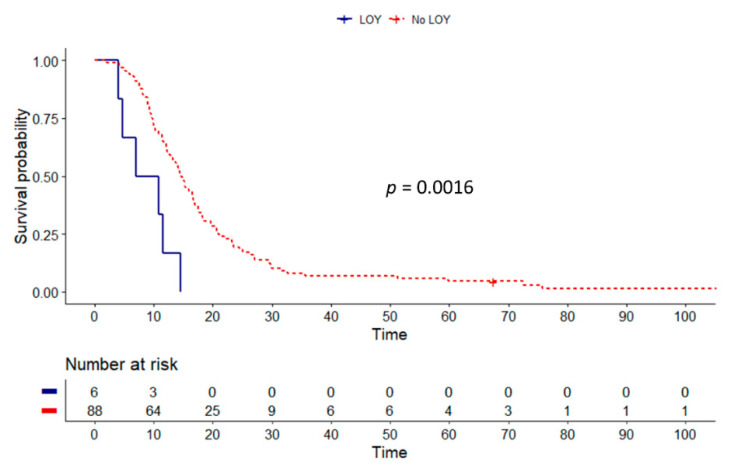
Kaplan–Meier log-rank survival analysis of patients with loss of chromosome Y (LOY) in GBM tumor tissue (*n* = 94). LOY in GBM (*n* = 6), defined as deletion of all 10 markers, correlates with shorter OS (7 vs. 14.5 months).

Then, we undertook survival analysis for individuals with a complete LOY. Indeed, these patients had significantly shorter OS (7 vs. 14.5 months, *p* = 0.002) (Figure 3); however, there were only six tumors with complete LOY (Figure 1b). No significant influence of deletion of the reported 3-gene cluster (ARSEP1, TMSB4Y, and UTY) on OS was observed (*p* > 0.05).

**Figure 4:**
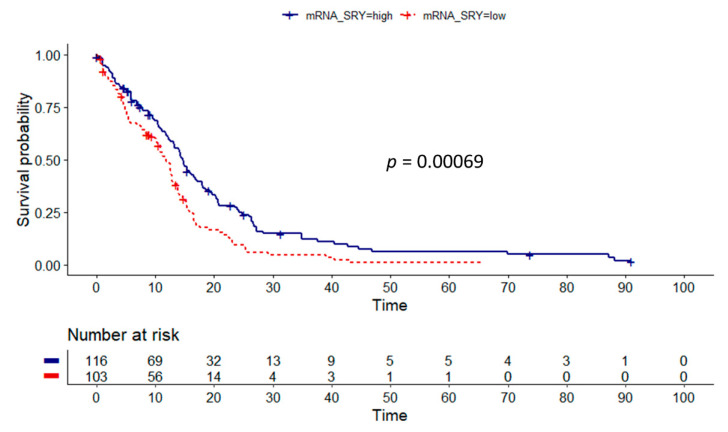
Kaplan–Meier log-rank survival analysis of low versus high SRY expressing GBM tumors (*n* = 219). Cut-off value was estimated via maximally selected rank statistics analysis (Supplementary Materials, Figure S2). Decreased gene expression correlates with shorter OS (11.9 vs. 14.7 months).

The TCGA cohort was examined similarly to the Swedish cohort, however the relative mRNA expression data of each gene was used as a proxy for CN aberrations. The best cut-off values, creating the largest differences in OS between low and high expression groups, were chosen through maximally selected rank statistics. We could analyze nine genes, as no mRNA expression data were available for the pseudogene ARSEP1, as expected. Out of the nine genes, six showed significant correlation of reduced mRNA expression to survival, while three (ZFY, KDM5D, and EIF1AY) did not reach statistical significance. The highest significance was noted for SRY, similarly to the CN analysis in the Swedish cohort. Patients with low expression of SRY (lower or equal to 3.3867(log2)) had shorter OS in comparison to the high expression group (11.9 vs. 14.7 months, *p* = 0.00069) (Figure 4). Significantly shorter OS associated with lower mRNA expression was also found for TMSB4Y (12.6 vs. 15.8 months, *p* = 0.0023), AMELY (12.7 vs. 14.2 months, *p* = 0.0087), and UTY (12.7 vs. 18.5 months, *p* = 0.0031). Surprisingly, in two cases low expression correlated with longer survival (USP9Y (14.1 vs.12.6 months, *p* = 0.039) and NLGN4Y (12.9 vs. 12.7 months, *p* = 0.039) (Supplementary Materials, Figure S2).

### 3.3. SRY Loss

**Figure 5:**
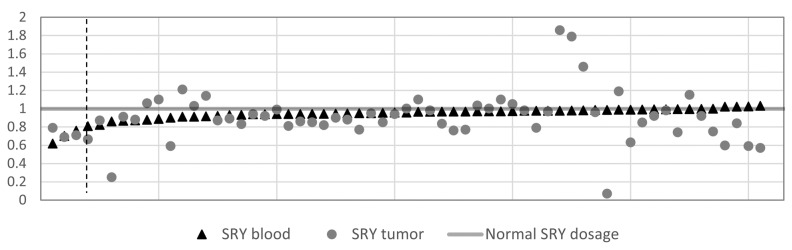
Comparison of SRY CN in the 61 matched blood and GBM samples. There is a large SRY CN divergence between both tissues, with most blood samples lacking any CN alterations. Thus, the CN aberrations can be considered tumor private. Dashed line separates 3 samples with SRY deletion (CN < 0.8) detected in both blood and tumor tissue.

Known chromosomal aberrations in tumors, as well as published research about Y chromosome loss in aging men, led to our finding regarding the impact of loss of SRY. This encouraged us to further study the nature of SRY deletions. Interestingly, the Mann–Whitney U tests did not show any significant age difference between males with the SRY deletion (mean age 56.8 years) and normal or elevated SRY CN (mean age 57.4 years), nor in TCGA group of patients with low (mean age 61.9 years) or high SRY expression (mean age 61.6 years). Additional CN analysis of SRY in the 61 tumor matched blood samples (Figure 5) revealed that only three tumor–blood pairs had deletions (CN ≤0.8) in both, indicating that SRY deletions are mainly tumor private.

Subsequently, we performed multivariate analysis of the Swedish cohort, which, apart from CN changes of SRY, also included known prognostic factors influencing OS in GBM: age, performance status, type of surgery, and MGMT promoter methylation status. The treatment regimen, concomitant chemoradiotherapy with temozolomide, was uniform for all patients, thus not a determinant, and therefore not included in the analysis. The Cox proportional hazard model created for the cohort showed an impact on OS for age (*p* = 0.017), MGMT status (*p* = 0.002), and to the greatest extent for deletion of SRY (*p* = 0.001) (Table 2).

Finally, we compared the relative SRY mRNA expression between GBM and lower grade gliomas (LGG grade II and III; mean age 37.9 years, range: 14–70 years) from TCGA. GBMs are generally characterized by a high expression of the SRY (mean relative expression 3.52(log2)), whereas LGG in many cases did not display any detectable expression, giving a negative mean expression value (−0.49) (Supplementary Materials, Figure S3). The difference was tested with the nonparametric Mann–Whitney U test and reached the significance level of 1.6 × 10^−41^.

### 3.4. Gene Set Enrichment Analysis

**Figure 6:**
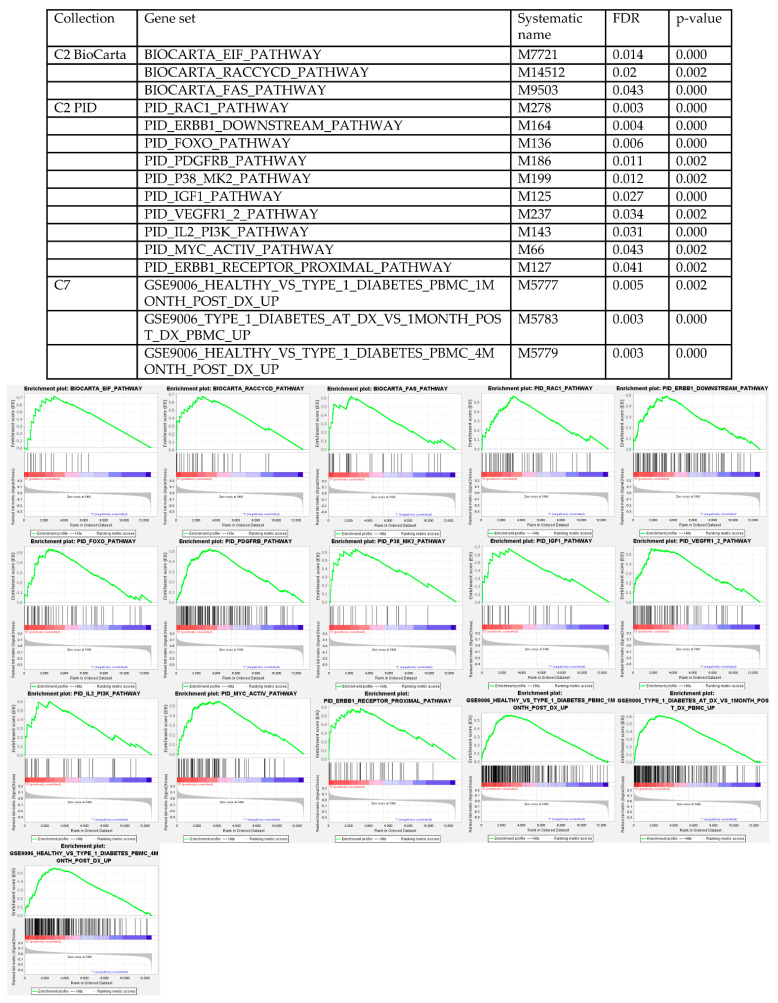
Gene Set Enrichment Analysis (GSEA) results. Table shows the gene sets found to be enriched in the low vs. high SRY expression group, with the FDR < 0.05 and *p*-value < 0.05. Enrichment plots are then presented for each of the sets.

To further explore the role of SRY in tumor development, we employed GSEA and tested TCGA gene expression data in all the major data set collections (H-C8) provided from the Molecular Signatures Database (MSigDB) [26]. We tested for sets enriched in the low vs. high SRY expressing tumors. Using the false discovery rate (FDR) limit of 0.05, only in three collections we were able to find enriched gene sets (Figure 6). Several sets were found in the collection containing canonical pathway lists curated from different databases (MSigDB C2 collection); three in the BioCarta and 10 in the Pathway Interaction Database collections. Additionally, three enriched gene sets were found in the collection containing immunologic signatures (MSigDB C7 collection). The emerging pathways found among the enriched gene sets in the low SRY expressing tumors implicated known major players of carcinogenesis, proliferation, cell migration, and apoptosis, e.g., MYC proto-oncogene (c-Myc), epidermal growth factor receptor family (ErbB) (with epidermal growth factor receptor (EGFR) being the most prominent), platelet-derived growth factor receptor (PDGFR), vascular endothelial growth factor receptor 1 and 2 (VEGFR1/2), phosphoinositide 3-kinase (PI3K)/protein kinase B (Akt), insulin like growth factor 1 (IGF1), eukaryotic translation initiation factor (eIF), Fas cell surface death receptor (Fas), Rac family small GTPase 1 (Rac1), and forkhead box O1 (FOXO) (Figure 6).

## 4. Discussion

In this study, we have shown that deletion of SRY, loss of genes located on chromosome Y, and complete LOY significantly influence survival of male GBM patients. Recognition of a sex bias in GBM has in recent years led to several studies addressing the underlying mechanisms of sex disparities, as reviewed by Matteoni et al. [27]. Different mutational burden [28], sex-dependent effects of IDH mutations [29] together with glycolytic dependency [30], and overrepresentation of Frizzled-7 receptor in the tumor cells [31] are only some of the proposed contributors. Surprisingly, sex chromosomes have not been studied as readily as the autosomal representatives, even though they comprise the obvious difference between the sexes. Therefore, we decided to study deletions on chromosome Y in GBM. Mosaic LOY has previously been linked with increased risk of non-hematological cancers, poor prognosis in head and neck squamous cell carcinoma, and/or all-cause mortality [12,13,32]. In addition, LOY has been shown to play a role in brain diseases, namely, Alzheimer’s disease [16] and schizophrenia [33]. Counterintuitively to the aforementioned studies, LOY also provides reduced risk of leukemic transformation in patients with myelodysplastic syndromes, but without this influencing survival [34].

The frequency of LOY in the male population varies depending on the studied subjects, affecting 8.2% in the primarily reported cohort of elderly men from the Uppsala Longitudinal Study of Adult Men [12] or as many as 28.6% in head and neck cancer [32]. In the present study, complete LOY was found in 6.4% of the samples with the full marker profile, placing this aberration in GBM on the lower end of the frequency spectrum. Despite the low percentage, LOY was strongly associated with shorter OS. As the relatively small sample size is a limitation of our study, it would be of interest to extend the analysis to additional cohorts.

The majority of studies of LOY [12,13,32,35] used the averaged signal value from the entire chromosome, which is a good approach for an overview. However, this approach eliminates the potential of identifying differences in a single gene or a group of genes localized on chromosome Y. For this reason, we analyzed the influence of each selected marker on OS separately as well as in combination. Similar to the methods applied by Mitchell et al. [18], we used probes across chromosome Y and an autosomal reference probe located on chromosome 5, which is seldom lost in brain tumors. From selected markers, eight out of ten have previously shown strong correlation between gene expression and gene dosage when tested with RNA-seq and Affymetrix genome-wide human SNP 6.0 arrays in TCGA of the head and neck squamous cell carcinomas data set [32]. We relied on the ddPCR method presented by Danielsson et al. [36] to be successful and consistent with the whole genome sequencing results when it comes to the Y chromosome. Moreover, the most recent study by Cácares et al. [35] showed a strong association between extreme downregulation of gene expression for genes present on chromosome Y, a proxy of LOY, and changes in the methylation patterns on chromosome Y, in different types of malignancies (e.g., glioma, colorectal cancer, and melanoma). The concept of LOY is widely discussed to comprise several biological layers that in combination contribute to a variety of diseases in the male population, as reviewed by Guo et al. [37]. Cácares et al. [35] presented a model based on TCGA data, in which cancer development is most likely an outcome of the extreme downregulation of chromosome Y, which in turn is a result of age dependent LOY.

In the analysis of CN in the Swedish cohort and TCGA gene expression, we found a correlation between deletion/loss of several genes on the Y chromosome and reduced OS. The one gene that consistently appeared to have the most significant prognostic influence is SRY. Interestingly, deletions of SRY had an even greater impact on survival than MGMT promoter methylation, a biomarker proven to be indispensable for the successful treatment with temozolomide [38]. Studied mainly for its sex-determining properties, SRY recently gained an unexpected label of a potential oncogene in hepatocellular carcinoma [39,40]. Ectopic expression of SRY in a transgenic mice model promoted hepatocarcinogenesis via activation of the SRY-box transcription factor 9 (Sox9), PDGFRα/PI3K/Akt, and c-Myc/CyclinD1 pathways [39]. The c-Myc activation was also shown to be an effect of SRY upregulating the SAGA complex associated factor 29 (Sgf29) expression, which takes part in histone H3 acetylation [40]. Through GSEA we could confirm that PDGFR, PI3K/Akt and c-Myc signaling pathways are linked to SRY in GBM. Surprisingly, these pathways were enriched in the low SRY expression group, contradicting findings from the hepatocellular carcinoma studies. One possible explanation for the discrepancy is that the modifying effects of ectopically expressed SRY are context-specific, depending on the availability of co-factors [41]. Thus, ectopic SRY expression in non-gonadal cells may compete with normal gene regulatory functions of the resident SRY-related HMG-box genes, encoding transcription factors, by disrupting the regulatory program [41]. Expression of SRY in LGG was almost undetectable, but these tumors have completely different genetic profiles in comparison to GBM [1], supporting the notion of context-specificity. Notably, SRY was shown to inhibit WNT/β-catenin signaling in the human pluripotent stem cells [42], and therefore low SRY expression may lead to proliferation, driven by upregulated WNT/β-catenin signaling in GBM [43]. Furthermore, GSEA revealed several of the GBM associated signaling pathways to be enriched in the low SRY expressing tumors, for example, EGFR, one of the key tumor regulators, aberrant especially in the classical subtype of GBM [44]. SRY contributes to the sex disparity observed in osteoporosis, downregulating expression of nuclear factor κB ligand (RANKL) [45], known also to promote invasiveness of glioblastoma cells [46]. Therefore, decreased expression of SRY could lead to the more aggressive tumors causing shortened OS. However, this relationship could not be confirmed in the TCGA cohort (SRY and RANKL Spearman’s correlation coefficient equal to 0.08, *p* > 0.05).

One could argue that variation in the dosage of genes detected in our study could be just a representation of genetic instability common in cancers [19]. This then being in line with our results from the tumor-matched blood samples showing that SRY deletions are more common in tumor tissue than in the blood of the GBM patients. Inclusion of only the primary, treatment-naïve tumors, diminishes the possibility of anticancer treatment influencing our results. LOY is recognized as an aberration increasingly appearing in the blood cells of aging men [12,13,17,19]; however, the frequency can fluctuate and even decrease after cessation of smoking or due to unknown factors [36]. Inconsistently, extreme downregulation of chromosome Y has been noted as a feature independent of age [35], similarly to our results, where loss of SRY and downregulated expression in tumor tissue were age independent. Unfortunately, smoking habits were not known for our patients. Our congruent findings from multiple sources do though support the significance of loss of SRY.

It should be addressed that the X chromosome’s involvement might be as important as participation of chromosome Y in tumorigenesis, even if mosaicism of chromosome X has not been associated with non-hematological cancer [47]. In fact, animal models show that Y-chromosomal homologous genes on the sex chromosomes are expressed in different cells of the developing male brain [48], which if occurring also in the adulthood, may represent a source of divergent behaviors in otherwise equal cells. Furthermore, neural stem cells respond differently to sex hormones and retinoic acid depending on the sex chromosome content [49], and, considering the stem cells in the subventricular zone as the precursors of GBM [50], there could exist a sex-driven susceptibility of neural stem cells to malignant transformation.

## 5. Conclusions

In summary, a growing body of evidence has indicated areas where a groundwork should be done to unravel mechanisms behind sex disparity in cancer, aligning such efforts with personalized medicine and warranting clinical trials focused on gender-specific treatment strategies. Our results support these strategies and show that SRY plays a significant role in GBM survival, a finding that warrants further studies on its functional role in gliomagenesis.

## Figures and Tables

**Table 1:** Clinicopathological data of male glioblastoma (GBM) patients from southeast Sweden.

Characteristics	Category	Number of Patients	Percentage
Age, years	≤60	64	61%
	>60	41	39%
Surgery	Biopsy	15	14%
	Partial resection	24	23%
	Large total resection	66	63%
Preoperative performance status WHO	0–1	83	79%
	2–3	19	18%
	Missing	3	3%
RT dose (Gray)	34 ^a^	7	6.7%
	40–50 ^b^	8	7.6%
	54–60	89	84.7%
	Missing	1	1%
MGMT	Methylated	36	65.7%
	Unmethylated	69	34.3%
SRY CN	≤0.6	12	11.4%
	>0.6	92	87.6%
	Missing	1	0.95%

^a^ Hypofractionated 3.4 Gy × 10; ^b^ RT stopped early.

**Table 2:** Multivariate analysis results. We included 104 samples with SRY CN data (for 1 sample we did not obtain the result) in the multivariate analysis and compared the influence of different prognostic factors on OS. Deletions of SRY have the most significant influence on OS.

Variables	Compared Groups	Hazard Ratio (95% Cl)	*p*-Value
Age	>60 vs. ≤60	1.702 (1.1–2.633)	0.017 *
Preoperative performance status (WHO)	2-3 vs. 0-1	0.937 (0.553–1.588)	0.810
Type of surgery	Biopsy/partial vs. large total	1.131 (0.736–1.740)	0.574
MGMT status	Unmethylated vs. methylated	2.071 (1.302–3.294)	0.002 *
CN of SRY	≤0.597 vs. >0.597	3.352 (1.695–6.629)	0.001 *

* *p* < 0.05.

## Data Availability

CN data from ddPCR analysis are available upon request and TCGA expression data are available via GlioVis portal as mentioned in the Methods section.

## Supplementary Materials

The following are available online at https://www.mdpi.com/article/10.3390/cancers13071619/s1, Supplementary Table S1: Clinicopathological data of male GBM patients from TCGA, Supplementary Table S2: Probes used in the ddPCR for estimation of CN of the genes on chromosome Y, Supplementary Figure S1: Results of the survival analysis based on the CN values with Kaplan–Meier log rank method after cut-off determination via maximally selected rank statistics (*p* < 0.05), Supplementary Figure S2: Results of the survival analysis based on the mRNA gene expression from TCGA with Kaplan–Meier log rank method after cut-off determination via maximally selected rank statistic, Supplementary Figure S3: Comparison of relative SRY expression between males with GBM and LGG from TCGA.

## Abbreviations

GBM	Glioblastoma
IDH	Isocitrate dehydrogenase
LOY	Loss of chromosome Y
TCGA	The Cancer Genome Atlas
MGMT	O-6-methylguanine-DNA methyltransferase
SRY	Sex determining region Y gene
ddPCR	Droplet digital PCR
OS	Overall survival
CN	Copy number
GSEA	Gene Set Enrichment Analysis
ZFY	Zinc finger protein Y-linked
AMELY	Amelogenin Y-linked
ARSEP1	Arylsulfatase L pseudogene 1
USP9Y	Ubiquitin specific peptidase 9 Y-Linked
UTY	Ubiquitously transcribed tetratricopeptide repeat containing, Y-Linked
TMSB4Y	Thymosin beta 4 Y-linked
NLGN4Y	Neuroligin 4 Y-linked
KDM5D	Lysine demethylase 5D
EIF1AY	Eukaryotic translation initiation factor 1A Y-linked
LGG	Lower grade gliomas
c-Myc	MYC proto-oncogene
ErbB	Epidermal growth factor receptor family
EGFR	Epidermal growth factor receptor
PDGFR	Platelet derived growth factor receptor
VEGFR1/2	Vascular endothelial growth factor receptor 1 and 2
PI3K	Phosphoinositide 3 kinase
Akt	Protein kinase B
IGF1	Insulin like growth factor 1
eIF	Eukaryotic translation initiation factor
Fas	Fas cell surface death receptor
Rac1	Rac family small GTPase 1
FOXO	Forkhead box O1
