# Supplementary material for: Deletions on Chromosome Y and Downregulation of the SRY Gene in Tumor Tissue Are Associated with Worse Survival of Glioblastoma Patients
Source: Cancers (Basel). 2021 Mar 31;13(7):1619. doi: 10.3390/cancers13071619 (PMC8036637; doi:10.3390/cancers13071619)
Supplement: Supplementary file 1 [file cancers-13-01619-s001.zip › Supplementary Materials.pdf]

Supplementary Table S1. Clinicopathological data of male GBM patients from TCGA.

		Number of patients	Percentage
Age, years	≤60	107	48%
	>60	118	52%
Treatment type	Alkylating/ TMZ chemo	6	2.7%
	TMZ chemoradiation + TMZ chemo	91	41.6%
	Standard radiation + alkylating/ TMZ chemo	52	23.7%
	Standard radiation	17	7.8%
	Nonstandard/ unspecified radiation	32	14.6%
	Nonstandard/ unspecified radiation + chemo (alkylating/ TMZ)	14	6.4%
	Unspecified therapy	7	3.2%

Supplementary Table S2. Probes used in the ddPCR for estimation of CN of the genes on chromosome Y.

Name of the gene	Location on chromosome Y	Name of the assay	Length [nucleotides]	Provider	Reference gene	Annealing temperature [˚C]
SRY	2,786,855-2,787,699	dHsaCP2500472	69	BioRad	AP3B1	60
ZFY	2,935,281-2,982,506	Hs05708066_cn	89	Applied Biosystems	AP3B1	60
AMELY	6,865,918-6,911,774	dHsaCNS670716622	77	BioRad	AP3B1	57
ARSEP1	12,339,813-12,347,495	dHsaCNS683350032	80	BioRad	AP3B1	57
USP9Y	12,537,650-12,860,839	dHsaCP2506328	69	BioRad	AP3B1	60
UTY	13,248,379-13,480,673	Hs0126180_cn	84	Applied Biosystems	AP3B1	55
TMSB4Y	13,703,899-13,706,024	dHsaCNS186618179	65	BioRad	AP3B1	57
NLGN4Y	14,522,573-14,845,650	Hs05704798_cn	85	Applied Biosystems	AP3B1	60
KDM5D	19,703,865-19,744,939	Hs02555392_cn	72	Applied Biosystems	AP3B1	55
EIF1AY	20,575,776-20,593,154	Hs01079143_cn	77	Applied Biosystems	AP3B1	55

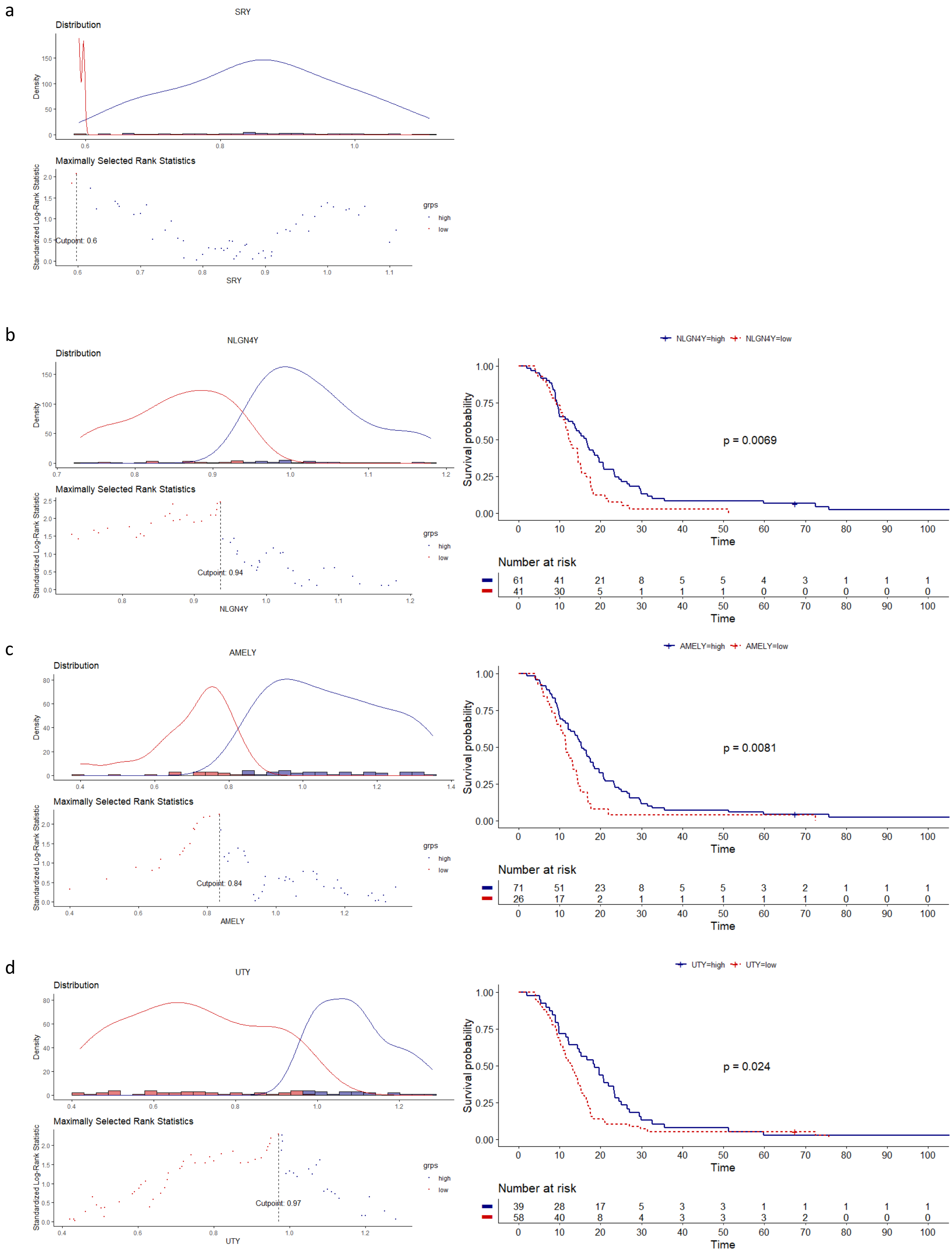
a
SRY
Distribution
Density
Maximally Selected Rank Statistics
Standardized Log-Rank Statistic
Cutpoint: 0.6
grps
high
low
SRY
b
NLGN4Y
Distribution
Density
Maximally Selected Rank Statistics
Standardized Log-Rank Statistic
Cutpoint: 0.94
grps
high
low
NLGN4Y
NLGN4Y=high
NLGN4Y=low
Survival probability
p = 0.0069
Time
Number at risk
61 41 21 8 5 5 4 3 1 1 1
41 30 5 1 1 1 0 0 0 0 0
Time
c
AMELY
Distribution
Density
Maximally Selected Rank Statistics
Standardized Log-Rank Statistic
Cutpoint: 0.84
grps
high
low
AMELY
AMELY=high
AMELY=low
Survival probability
p = 0.0081
Time
Number at risk
71 51 23 8 5 5 3 2 1 1 1
26 17 2 1 1 1 1 1 0 0 0
Time
d
UTY
Distribution
Density
Maximally Selected Rank Statistics
Standardized Log-Rank Statistic
Cutpoint: 0.97
grps
high
low
UTY
UTY=high
UTY=low
Survival probability
p = 0.024
Time
Number at risk
39 28 17 5 3 3 1 1 1 1 1
58 40 8 4 3 3 3 2 0 0 0
Time

e

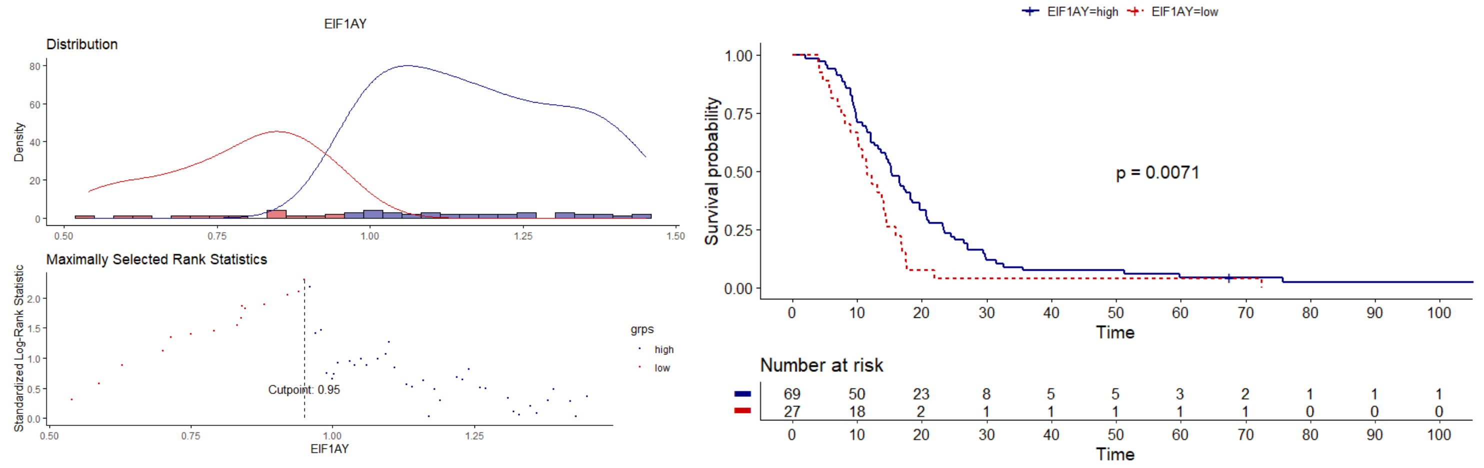
EIF1AY
Distribution
Density
Maximally Selected Rank Statistics
Standardized Log-Rank Statistic
Cutpoint: 0.95
grps
high
low
EIF1AY
EIF1AY=high
EIF1AY=low
Survival probability
p = 0.0071
Time
Number at risk
69 50 23 8 5 5 3 2 1 1 1
27 18 2 1 1 1 1 1 0 0 0
0 10 20 30 40 50 60 70 80 90 100
Time

f

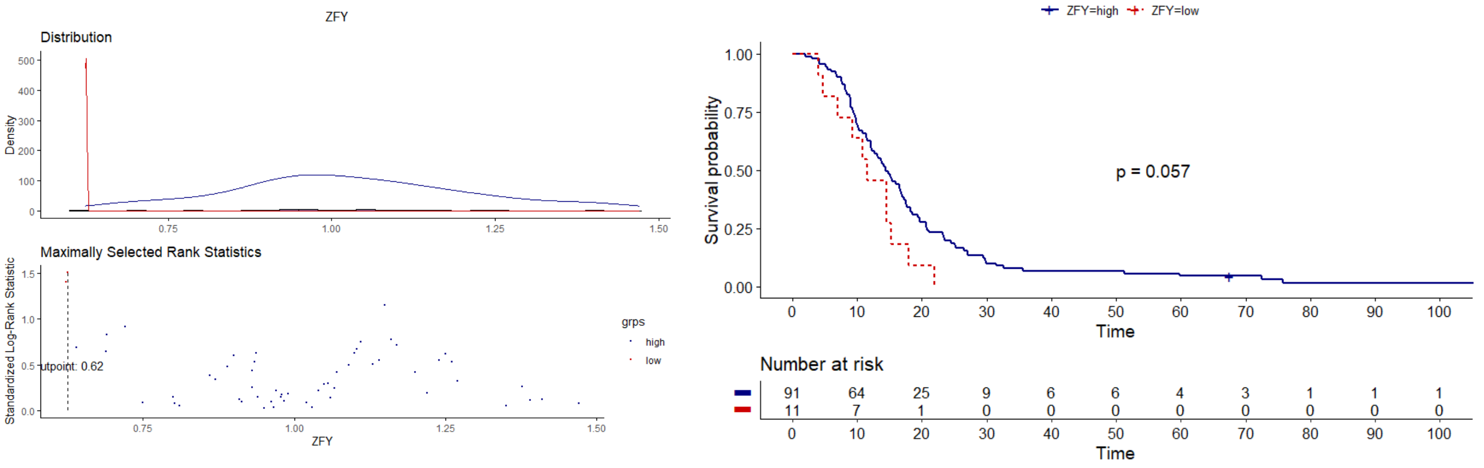
ZFY
Distribution
Density
Maximally Selected Rank Statistics
Standardized Log-Rank Statistic
Cutpoint: 0.62
grps
high
low
ZFY
ZFY=high
ZFY=low
Survival probability
p = 0.057
Time
Number at risk
91 64 25 9 6 6 4 3 1 1 1
11 7 1 0 0 0 0 0 0 0 0
0 10 20 30 40 50 60 70 80 90 100
Time

g

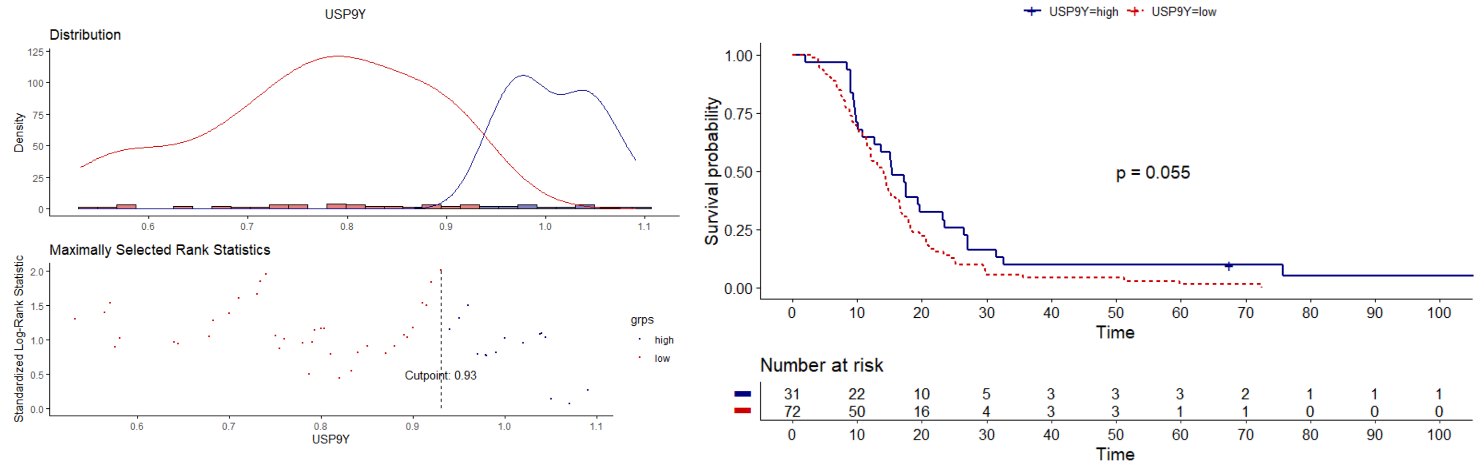
USP9Y
Distribution
Density
Maximally Selected Rank Statistics
Standardized Log-Rank Statistic
Cutpoint: 0.93
grps
high
low
USP9Y
USP9Y=high
USP9Y=low
Survival probability
p = 0.055
Time
Number at risk
31 22 10 5 3 3 3 2 1 1 1
72 50 16 4 3 3 1 1 0 0 0
0 10 20 30 40 50 60 70 80 90 100
Time

h

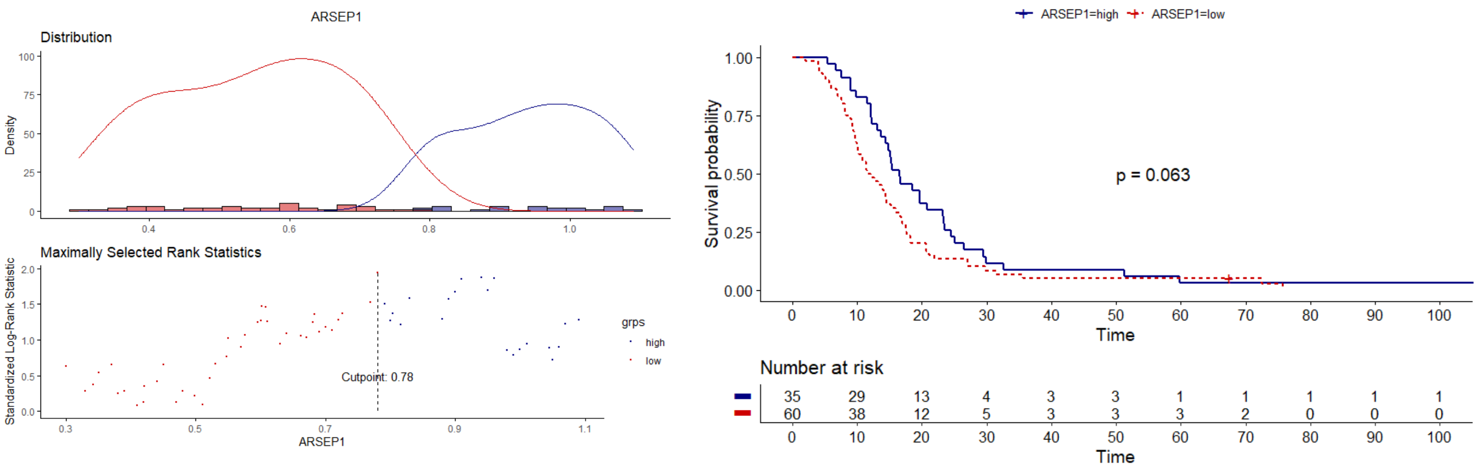
ARSEP1
Distribution
Density
Maximally Selected Rank Statistics
Standardized Log-Rank Statistic
Cutpoint: 0.78
grps
high
low
ARSEP1
ARSEP1=high
ARSEP1=low
Survival probability
p = 0.063
Time
Number at risk
35 29 13 4 3 3 1 1 1 1 1
60 38 12 5 3 3 3 2 0 0 0
0 10 20 30 40 50 60 70 80 90 100
Time

i

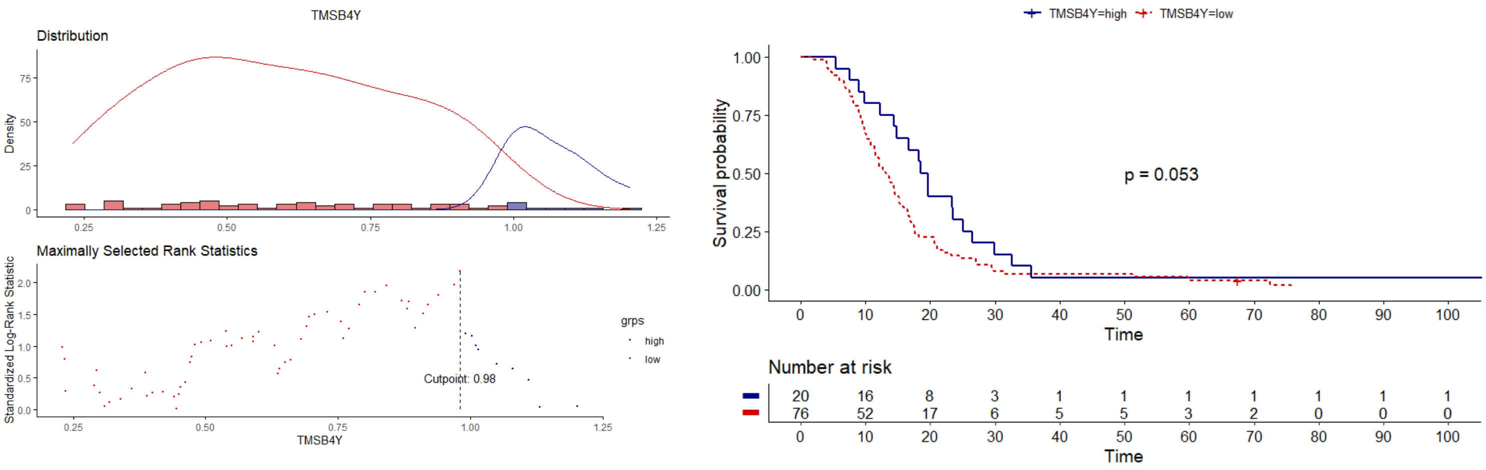

j

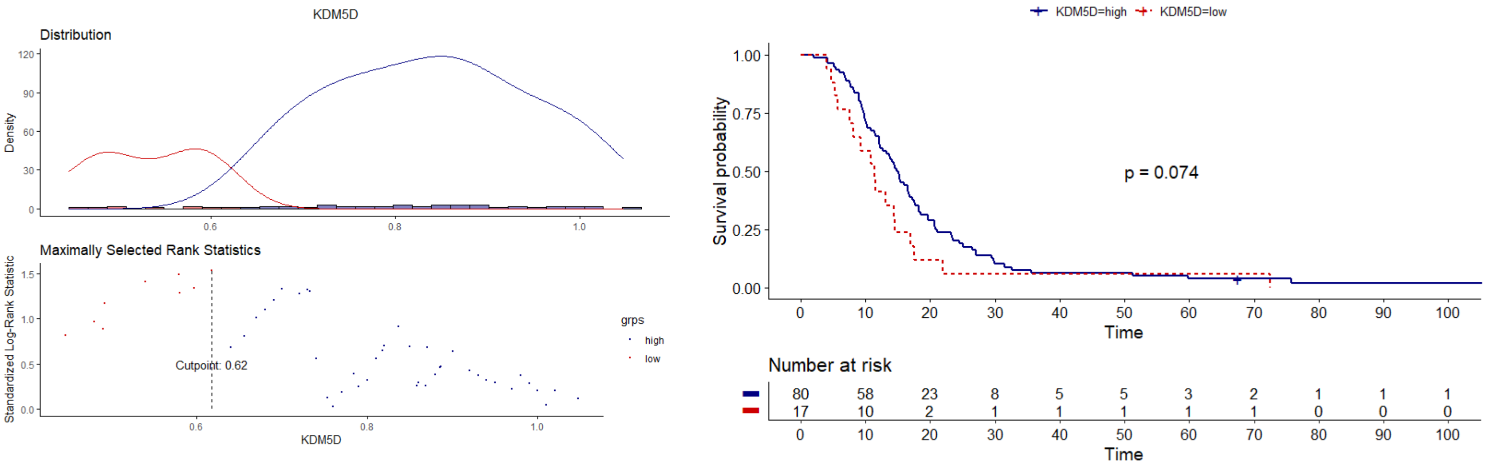

Supplementary Figure S1. Results of the survival analysis based on the CN values with Kaplan-Meier log rank method after cut-off determination via maximally selected rank statistics ($p<0.05$). On the left, graphs for selection of the best cut-off point, on the right, survival curves ((a) *SRY*; (b) *NLGN4Y*; (c) *AMELY*; (d) *UTY*; (e) *EIF1AY*; (f) *ZFY*; (g) *USP9Y*; (h) *ARSEP1*; (i) *TMSB4Y*; (j) *KDM5D*).

a

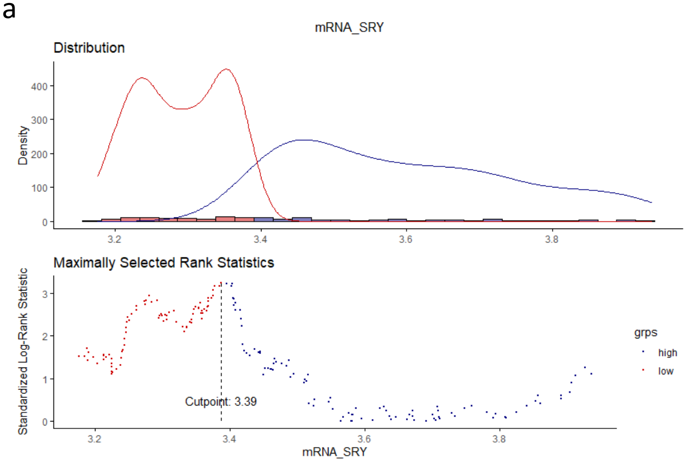
mRNA_SRY
Distribution
Density
Maximally Selected Rank Statistics
Standardized Log-Rank Statistic
Cutpoint: 3.39
mRNA_SRY
grps
high
low

b

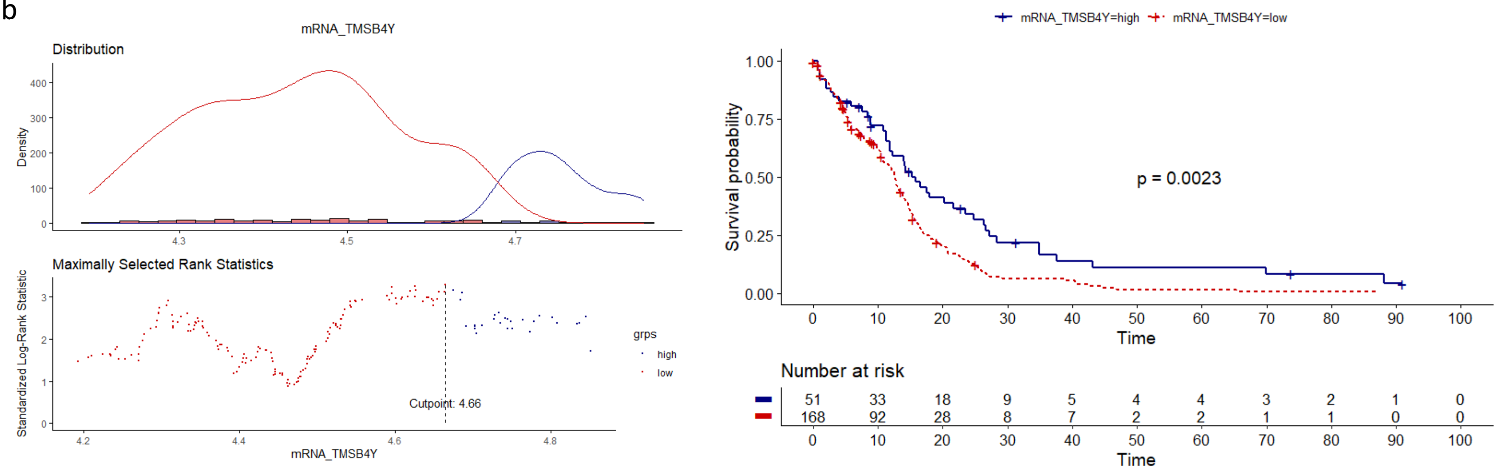
mRNA_TMSB4Y
Distribution
Density
Maximally Selected Rank Statistics
Standardized Log-Rank Statistic
Cutpoint: 4.66
mRNA_TMSB4Y
grps
high
low
mRNA_TMSB4Y=high
mRNA_TMSB4Y=low
Survival probability
p = 0.0023
Time
Number at risk
51 33 18 9 5 4 4 3 2 1 0
168 92 28 8 7 2 2 1 1 0 0

c

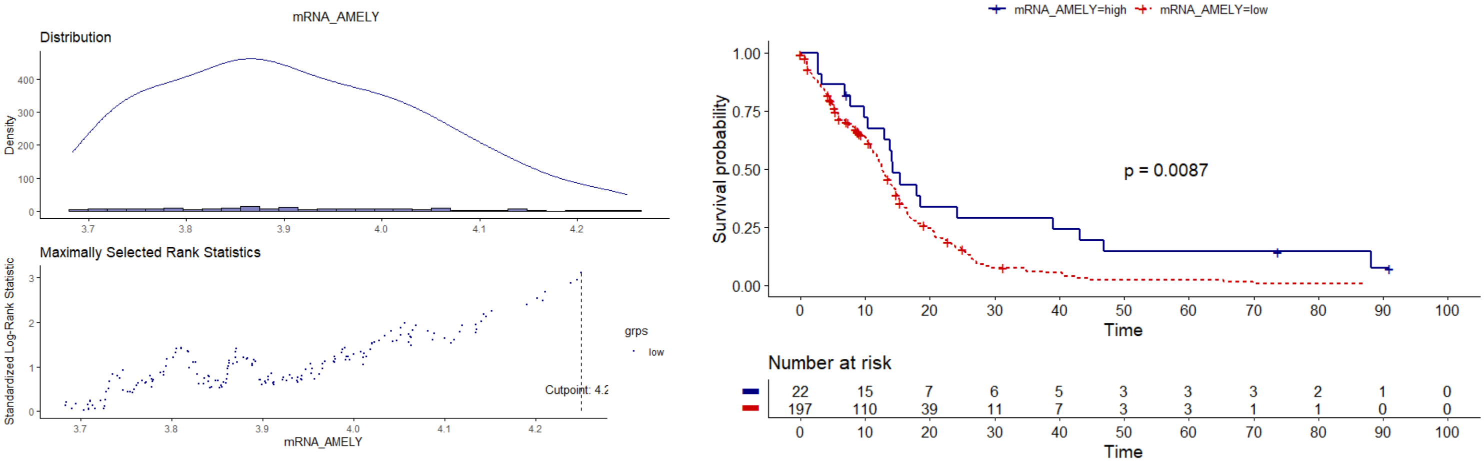
mRNA_AMELY
Distribution
Density
Maximally Selected Rank Statistics
Standardized Log-Rank Statistic
Cutpoint: 4.2
mRNA_AMELY
grps
low
mRNA_AMELY=high
mRNA_AMELY=low
Survival probability
p = 0.0087
Time
Number at risk
22 15 7 6 5 3 3 3 2 1 0
197 110 39 11 7 3 3 1 1 0 0

d

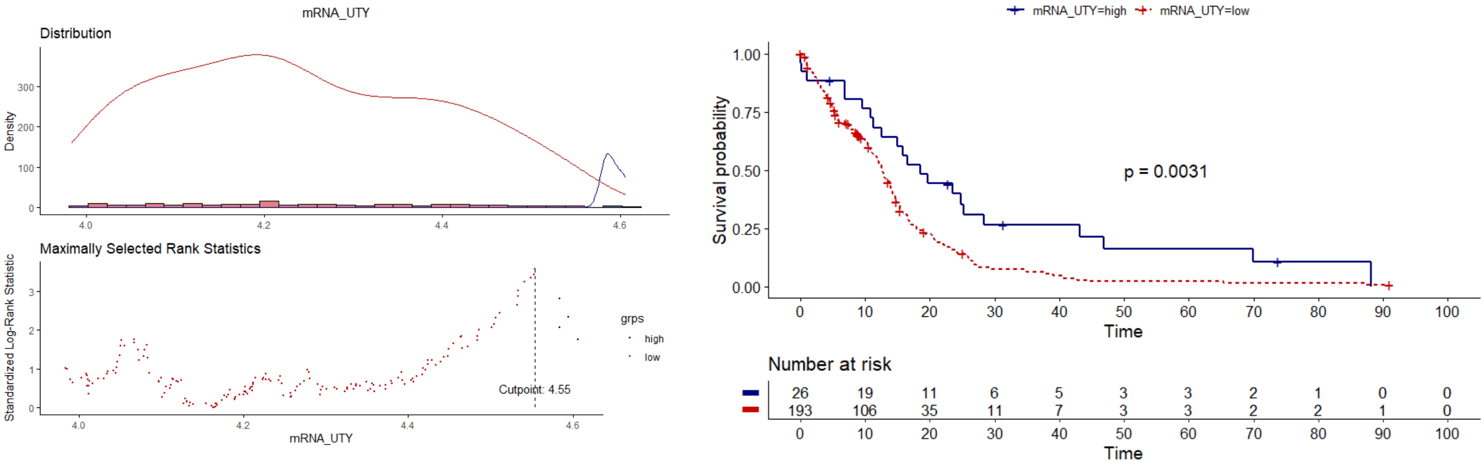
mRNA_UTY
Distribution
Density
Maximally Selected Rank Statistics
Standardized Log-Rank Statistic
Cutpoint: 4.55
mRNA_UTY
grps
high
low
mRNA_UTY=high
mRNA_UTY=low
Survival probability
p = 0.0031
Time
Number at risk
26 19 11 6 5 3 3 2 1 0 0
193 106 35 11 7 3 3 2 2 1 0

e

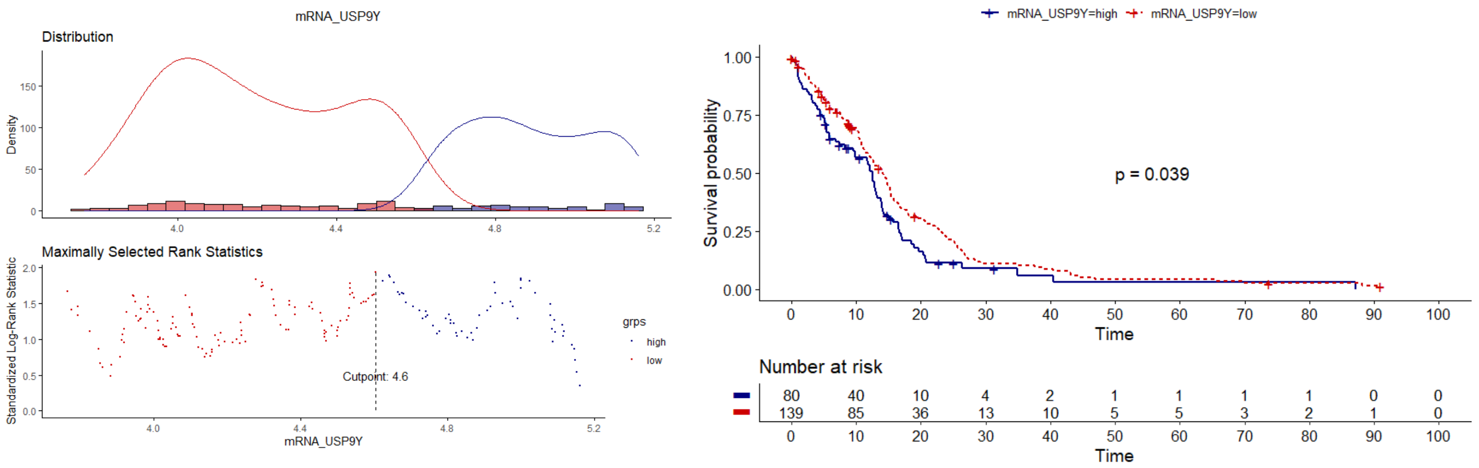
mRNA_USP9Y
Distribution
Density
Maximally Selected Rank Statistics
Standardized Log-Rank Statistic
Cutpoint: 4.6
grps
high
low
mRNA_USP9Y
mRNA_USP9Y=high
mRNA_USP9Y=low
Survival probability
p = 0.039
Time
Number at risk
80 40 10 4 2 1 1 1 1 0 0
139 85 36 13 10 5 5 3 2 1 0
Time

f

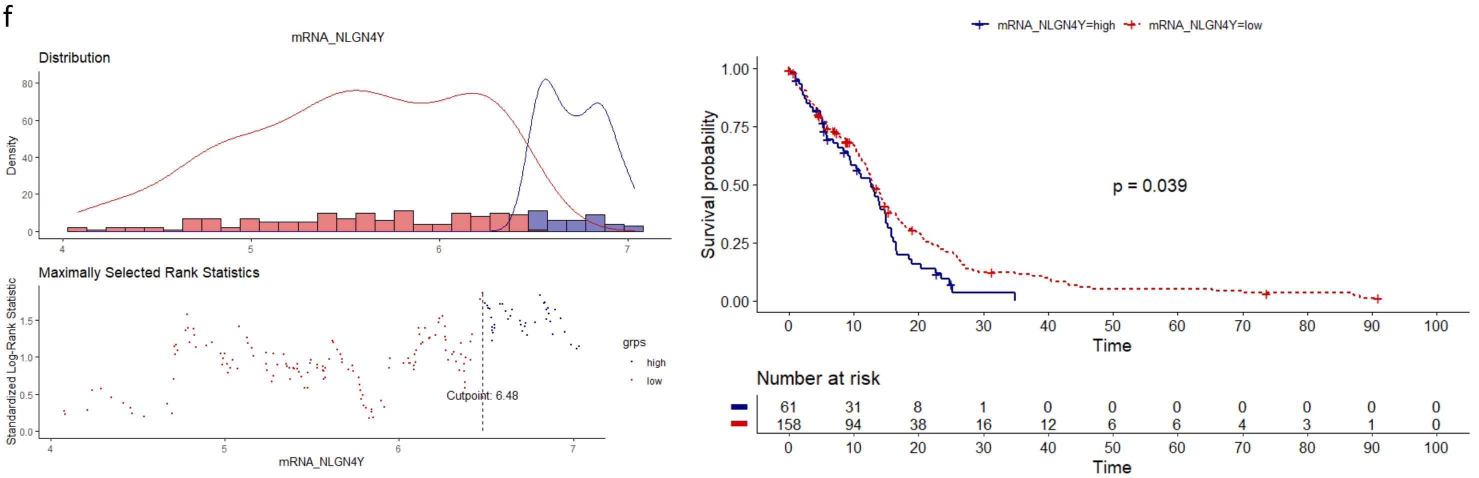
mRNA_NLGN4Y
Distribution
Density
Maximally Selected Rank Statistics
Standardized Log-Rank Statistic
Cutpoint: 6.48
grps
high
low
mRNA_NLGN4Y
mRNA_NLGN4Y=high
mRNA_NLGN4Y=low
Survival probability
p = 0.039
Time
Number at risk
61 31 8 1 0 0 0 0 0 0 0
158 94 38 16 12 6 6 4 3 1 0
Time

g

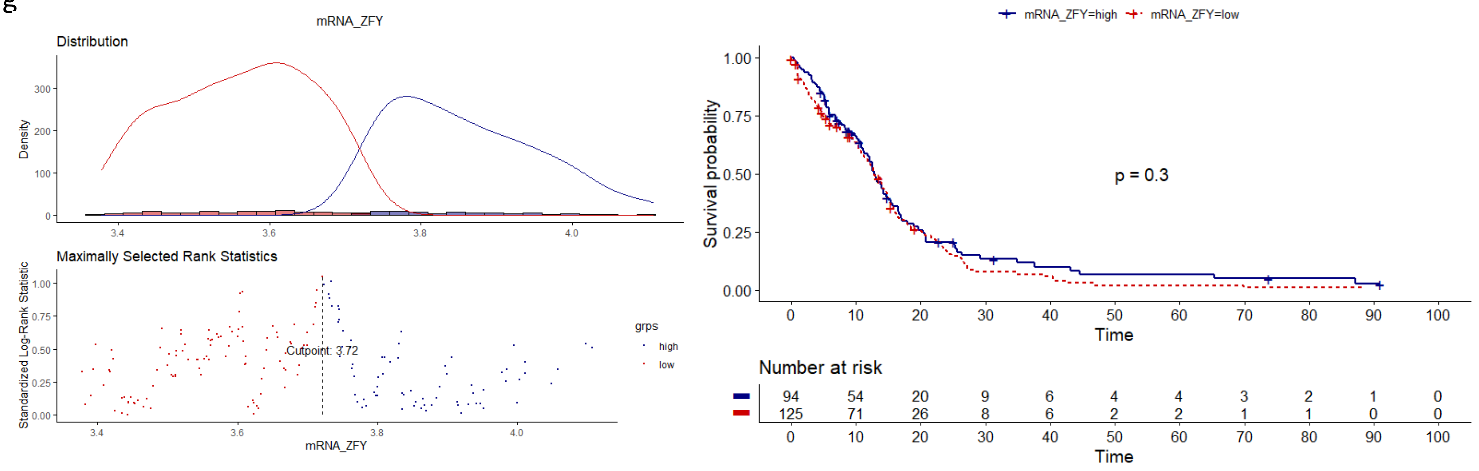
mRNA_ZFY
Distribution
Density
Maximally Selected Rank Statistics
Standardized Log-Rank Statistic
Cutpoint: 3.72
grps
high
low
mRNA_ZFY
mRNA_ZFY=high
mRNA_ZFY=low
Survival probability
p = 0.3
Time
Number at risk
94 54 20 9 6 4 4 3 2 1 0
125 71 26 8 6 2 2 1 1 0 0
Time

h

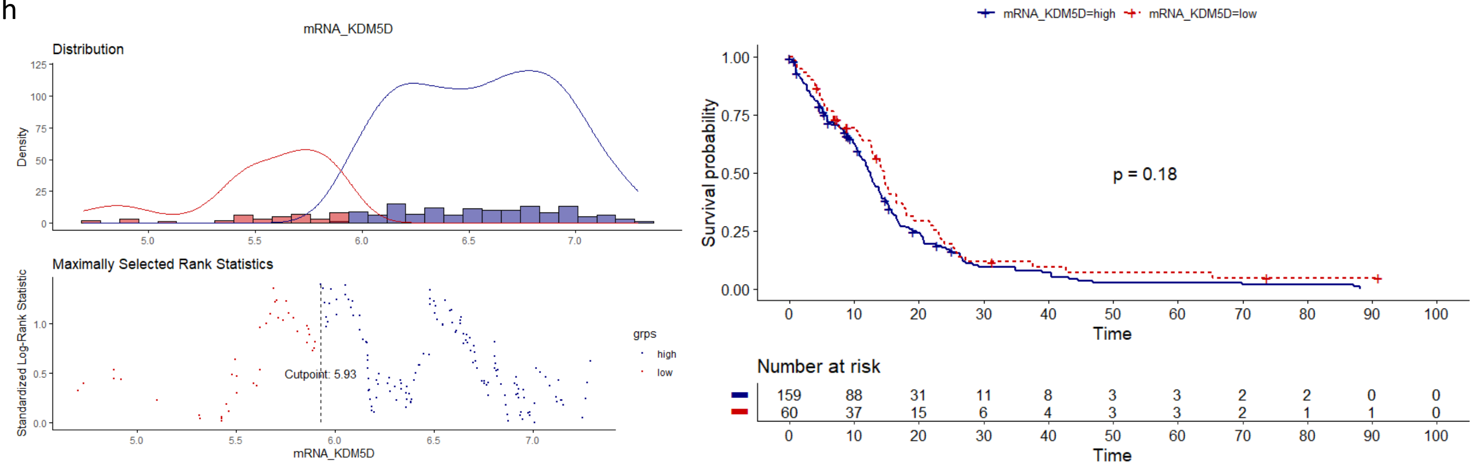
mRNA_KDM5D
Distribution
Density
Maximally Selected Rank Statistics
Standardized Log-Rank Statistic
Cutpoint: 5.93
grps
high
low
mRNA_KDM5D
mRNA_KDM5D=high
mRNA_KDM5D=low
Survival probability
p = 0.18
Time
Number at risk
159 88 31 11 8 3 3 2 2 0 0
60 37 15 6 4 3 3 2 1 1 0
Time

i

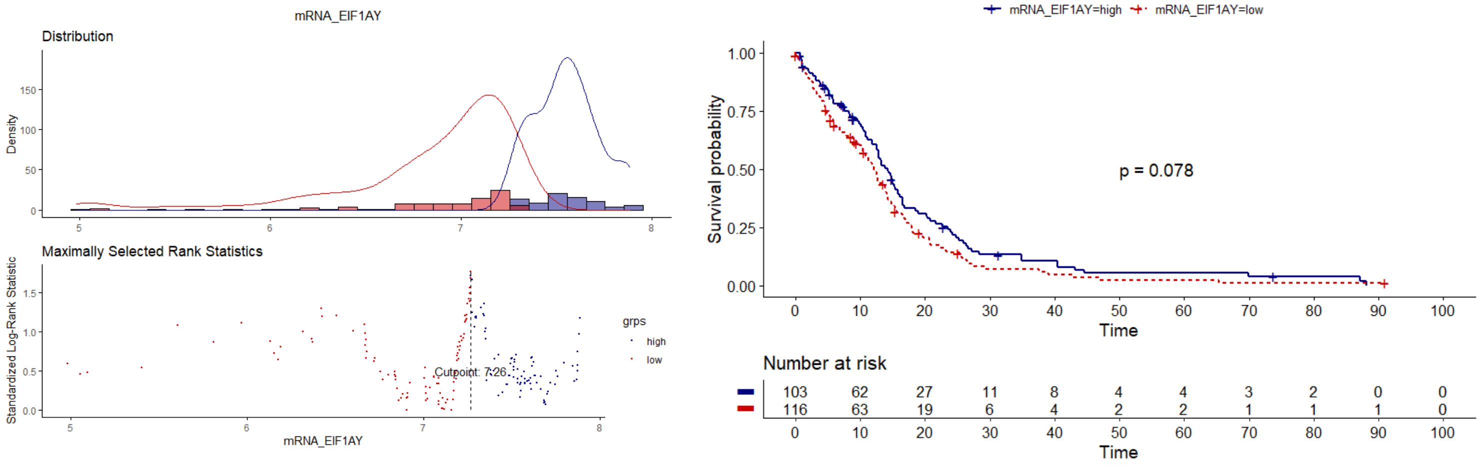

Supplementary Figure S2. Comparison of relative *SRY* expression between males with GBM and LGG from TCGA. LGG have very low *SRY* expression, nearly undetectable, whereas GBM can be characterized as high *SRY*-expressing tumor. The difference was tested with the non-parametric Mann-Whitney U test and is statistically significant ($p=1.6 \times 10^{-41}$).

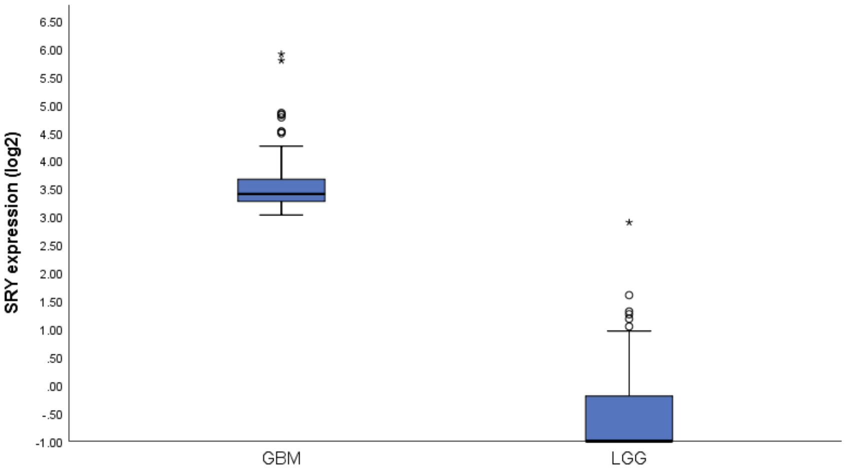

Supplementary Figure S3. Results of the survival analysis based on the mRNA gene expression from TCGA with Kaplan-Meier log rank method after cut-off determination via maximally selected rank statistic. On the left, graphs for selection of the best cut-off point, on the right, Kaplan-Meier survival curves. (a) cut-off determination for the most significant gene *SRY;* (b-f) genes with significant influence on the OS of GBM male patients; (e, f) statistically significant results where lower expression of genes correlated with shorter OS; (g-i) genes without significant influence on the OS.
